# Differential Sensitivity to *Plasmodium yoelii* Infection in C57BL/6 Mice Impacts Gut-Liver Axis Homeostasis

**DOI:** 10.1038/s41598-019-40266-6

**Published:** 2019-03-05

**Authors:** Joshua E. Denny, Joshua B. Powers, Hector F. Castro, Jingwen Zhang, Swati Joshi-Barve, Shawn R. Campagna, Nathan W. Schmidt

**Affiliations:** 1 Department of Microbiology and Immunology, University of Louisville, Louisville, KY 40202 USA; 2 Department of Chemistry, University of Tennessee, Knoxville, TN 37996 USA; 3 Department of Medicine; Alcohol Research Center; and Hepatobiology and Toxicology Center, University of Louisville, Louisville, Kentucky 40202 USA

## Abstract

Experimental models of malaria have shown that infection with specific *Plasmodium* species in certain mouse strains can transiently modulate gut microbiota and cause intestinal shortening, indicating a disruption of gut homeostasis. Importantly, changes in gut homeostasis have not been characterized in the context of mild versus severe malaria. We show that severe *Plasmodium* infection in mice disrupts homeostasis along the gut-liver axis in multiple ways compared to mild infection. High parasite burden results in a larger influx of immune cells in the lamina propria and mice with high parasitemia display specific metabolomic profiles in the ceca and plasma during infection compared to mice with mild parasitemia. Liver damage was also more pronounced and longer lasting during severe infection, with concomitant changes in bile acids in the gut. Finally, severe *Plasmodium* infection changes the functional capacity of the microbiota, enhancing bacterial motility and amino acid metabolism in mice with high parasite burden compared to a mild infection. Taken together, *Plasmodium* infections have diverse effects on host gut homeostasis relative to the severity of infection that may contribute to enteric bacteremia that is associated with malaria.

## Introduction

Malaria infections, caused by *Plasmodium*, have long been of global clinical importance, with 216 million infections and approximately 445,000 deaths in 2016^1^. Several factors play a role in disease epidemiology, such as emerging resistance to frontline antimalarials^2,3^ and the lack of an effective vaccine. Further complicating efforts to eradicate this parasite is the dual life cycle wherein *Plasmodium* sexually reproduces and develops in its reservoir, the *Anopheles* mosquito, before being transmitted to the human host during a mosquito blood meal^4,5^. Within the human host the parasite undergoes development in the liver followed by asexual reproduction in red blood cells^4,5^. The liver stage is clinically silent, while the blood stage is associated with the clinical symptoms of malaria, including fever, anemia, and coma^4,5^.

The microbiota, which is the microbial consortia associated with the host, has been connected to host homeostasis and development. Bacteria are the most common inhabitants, while fungi and archaea make up smaller parts of the consortia. Indeed, recent calculations estimate there are approximately 3–4 × 10^13^ associated bacteria, corresponding to a bacteria:human cell ratio from around 1.3 to 2.3 depending on variables such as gender, age, and obesity^6^. The host microbiota has been examined in different sites such as skin^7,8^ and lung^9–11^, but the gut microbiota has by far been the most studied. Intriguingly, the gut microbiota has also been shown to interact with the central nervous system in the “gut-brain axis”, and is involved in processes like host development, circadian rhythm, and disease states such as major depression^12,13^. The gut microbiota has been shown to play a role in immunity, both locally in the intestine but also systemically in modulating host responses to diseases such as influenza and *Klebsiella pneumonia*^14–17^.

Likewise, it has previously been shown in humans and experimental mouse models that *Plasmodium* infection and the severity of malaria can be modulated by the composition of gut microbiota. Antibody cross-reactivity with the gut commensal *E. coli* O86:B7 and the expressed malaria antigen Galα1-3Galβ1-4GlcNAc-R (alpha-gal) leads to inhibition of sporozoite transmission through the skin^18^. We have previously observed that gut microbiota composition in mice can modulate the severity of *P. yoelii* 17XNL (Py) infection, and that susceptibility or resistance can be transferred to germ-free mice by transferring cecal contents from either susceptible or resistant mice^19^. Conversely, the *Plasmodium* infections also affect gut microbiota. Following *Plasmodium berghei* ANKA infection in C57BL/6 mice, which causes experimental cerebral malaria in C57BL/6 mice, gut microbiota became dysbiotic, or disrupted, as the infection progressed^20^. The authors concluded that the change in microbiota composition was due to *P. berghei* ANKA infection and not infection-associated inflammation, as the microbiota changes occurred before the intestines underwent inflammation-mediated changes such as the intestine and villi shortening^20^. In contrast, there were little to no changes in gut bacteria observed in *P. berghei* ANKA infected BALB/c mice^20^. Finally, C57BL/6 mice infected with *Plasmodium yoelii nigeriensis* also exhibited changes in gut microbiota composition during peak infection, although these changes were transient with the composition returning to baseline following resolution of the infection^21^.

While it has been shown that gut microbiota affects and is affected by *Plasmodium* infection, these findings have not been explored in the context of mild vs. severe malaria. Using a mouse model of malaria, we show that severe malaria differentially disrupts gut homeostasis compared to a mild infection. Severe Py infection leads to more proinflammatory cell infiltration in the intestinal lamina propria, as well as differential metabolic changes during infection. Severe infection also leads to prolonged liver damage; surprisingly, mild Py infection led to longer-lasting changes in cecal bile acid abundances. Following infection there were shifts in the taxonomy of gut bacteria in both mild and severe Py infections, with the composition of the gut bacteria becoming more similar over the course of infection. Of note, these changes did not impact the severity of malaria in subsequent infections. Finally, severe infection drives a differential functional profile in the gut microbiota compared to mild infection. These results show that severe Py infection can differentially disrupt gut homeostasis in numerous ways.

## Results

### Intestinal Permeability Increases During Py Infection but is not a Function of Parasite Burden

**Figure 1:**
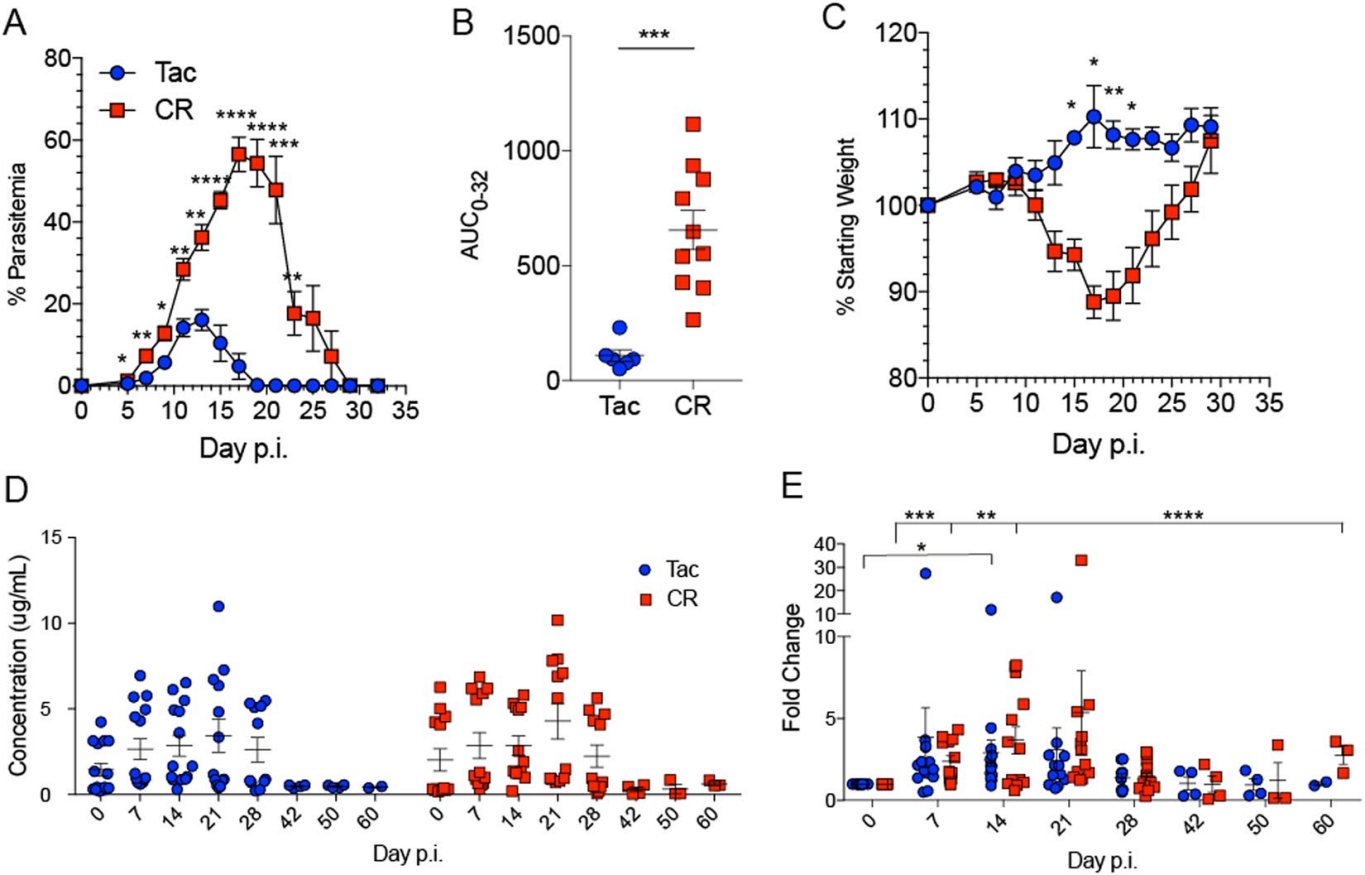
Susceptibility to *Plasmodium* infection varies between vendors. C57BL/6 N mice from Taconic (Tac) and Charles River (CR) were infected with *P. yoelii* (Py). (**A**) Percent parasitemia (percentage of red blood cells (RBCs) infected with Py) over the course of infection. Individual time points between Tac and CR were analyzed by unpaired two-tailed t-test. (**B**) Area under the curve (AUC) analysis of percent parasitemia. Data were analyzed by unpaired two-tailed t-test. (**C**) Percentage of weight lost post-infection (p.i.). Individual time points were analyzed by unpaired two-tailed t-test. (**D**) FITC-dextran concentrations in serum over the course of infection. (**E**) Data from panel D normalized to the Day 0 time point to show fold changes. All data were analyzed by unpaired two-tailed t-test. (**A**–**C**) Data (mean ± SE) are cumulative results (n = 3–5 mice/group) of two experiments. (**D**,**E**) Data (mean ± SE) are cumulative results of 3 experiments (n = 4–5 mice/group/experiment). *p < 0.05; **p < 0.01; ***p < 0.001; ****p < 0.0001.

During Py infection, C57BL/6 N mice from Taconic Biosciences (Tac) and Charles River Laboratories (CR) show different parasitemia kinetics, leading to significantly different overall parasite burdens with CR mice exhibiting higher parasitemia than Tac mice (Fig. 1A,B). CR mice also display more weight loss, indicating greater morbidity during infection (Fig. 1C). These characteristics allow us to contrast a relatively mild Py infection in Tac mice to a severe Py infection in CR mice and reaffirm the phenotypes we have observed previously^19^. We also looked at intestinal permeability during infection as one factor in gut homeostasis. While there were increases in intestinal permeability within the Tac and CR groups during infection, there were no differences between Tac and CR (Fig. 1D,E) indicating that increased intestinal permeability is not a function of overall parasite burden.

### The Lamina Propria Immune System Undergoes Differential Changes During Mild and Severe Py Infections

**Figure 2:**
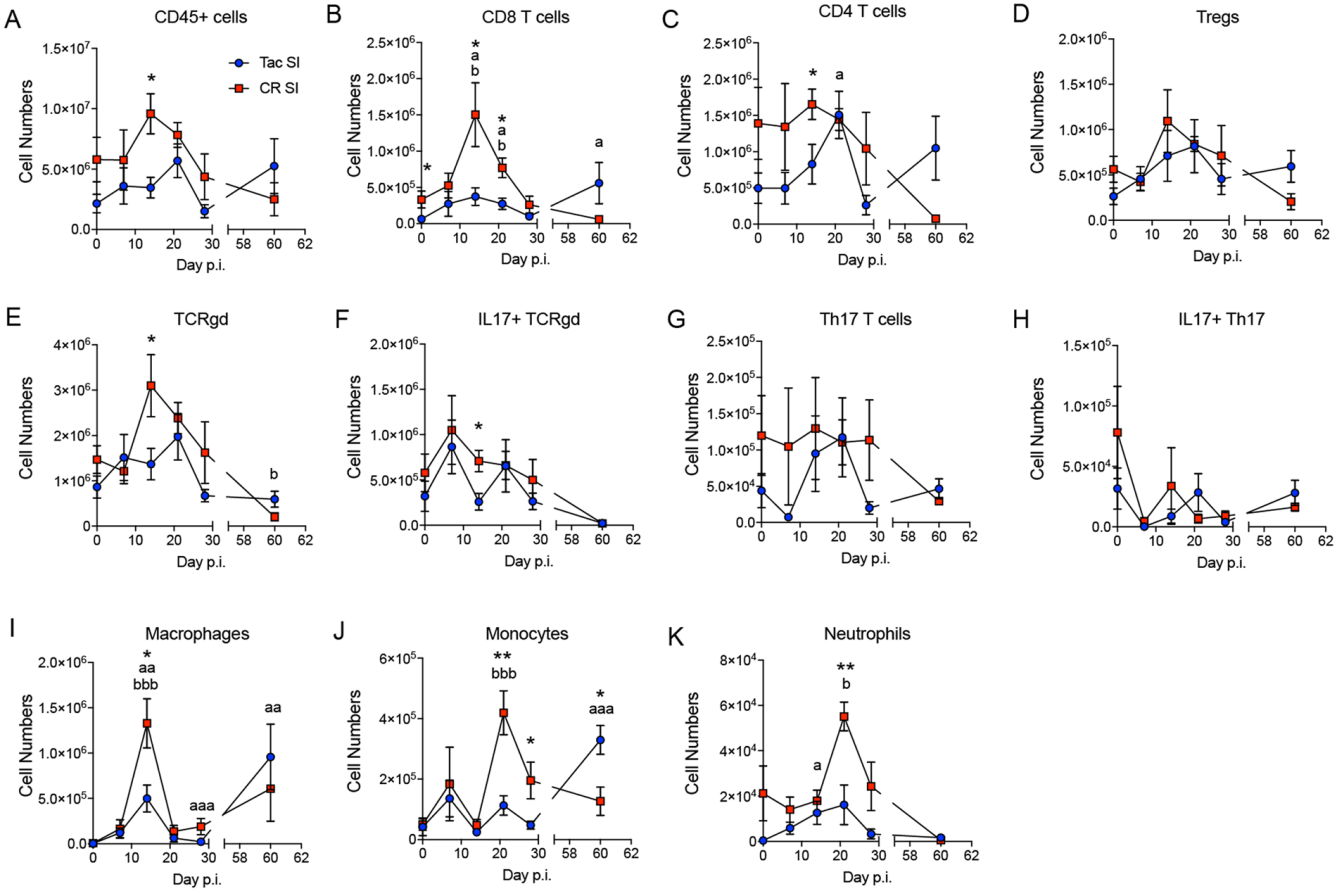
Small intestine lamina propria immune system changes during Py infection. Total cell numbers of (**A**) CD45+ cells, (**B**) CD8+ T cells, (**C**) CD4+ T cells, (**D**) Tregs, (**E**) Gamma delta T cells (TCRgd), (**F**) IL17+ TCRgd (**G**) Th17 cells, (**H**) IL17+ Th17 cells, (**I**) Macrophages, (**J**) Monocytes, and (**K**) Neutrophils. Each time point was compared by one-way ANOVA with Tukey’s post-hoc multiple comparison test. Data (mean ± SE) are cumulative results of 2 experiments (3 mice/group/experiment). 1 symbol, p < 0.05; 2 symbols, p < 0.01; 3 symbols, p < 0.001; 4 symbols, p < 0.0001. *Tac and CR SI comparisons; ^a^Tac SI comparisons to Day 0; ^b^CR SI comparisons to Day 0.

The lamina propria (LP) houses a very diverse immune cell population responsible for maintaining tolerance to gut microbiota^22–24^. Additionally, it has been shown that a systemic infection like influenza can modulate the LP immune system^14,15^. With this in mind, we followed specific immune cell populations in both the small intestine (SI) LP and large intestine (LI) LP during Py infection. In general, CR mice had significantly more immune cells in the SI LP during infection than Tac mice (Fig. 2 and Supplementary Fig. 1), particularly as the infection progressed and parasitemia peaked around days 14–21 post-infection (p.i.). More specifically, macrophages (CD45^+^SiglecF^−^CD11b^+^Ly6G^−^Ly6C^−^F4/80^+^) and CD8 T cells (CD45^+^CD8^+^) peaked at day 14 (Fig. 2B,I; Supplementary Fig. 1), while monocytes (CD45^+^SiglecF^−^CD11b^+^Ly6G^−^Ly6C^+^) and neutrophils (CD45^+^SiglecF^−^CD11b^+^Ly6G^+^) peaked at day 21 p.i. in CR mice (Fig. 2J,K; Supplementary Fig. 1). The influx of monocytes and neutrophils indicates a more inflammatory LP environment for CR mice at the peak of Py infection, which could cause changes in gut microbiota. Tac mice similarly had an increase in CD8 T cells and macrophages at day 14, along with a day 14 increase in neutrophils that correlate with peak parasitemia in Tac mice (Fig. 2B and I,J). Additionally, after Py clearance in the Tac SI: CD8 T cells (Fig. 2B), macrophages (Fig. 2I), and monocytes (Fig. 2J) all increased at day 60 p.i. In the LI LP (Supplementary Fig. 2), there were fewer changes than in the SI LP: macrophages increased in Tac and CR LI at day 14 and day 60 p.i. (Supplementary Fig. 2I) and IL17 + Th17 T cells (Supplementary Fig. 2H) increased in both Tac and CR mice after Py clearance. While both Tac and CR had a significant increase in IL17 + gamma delta T cells (TCRgd; CD45^+^TCRgd^+^IL17^+^) at days 7 and 14 p.i., Tac mice had more than CR at day 14 (Supplementary Fig. 2F). Overall, distinct immune populations change during mild and severe malaria, with a potentially more inflammatory environment during severe malaria in CR mice.

### Gut Microbiota Undergoes Significant Post-Infection Changes but does not Affect Susceptibility to Future Infections

**Figure 3:**
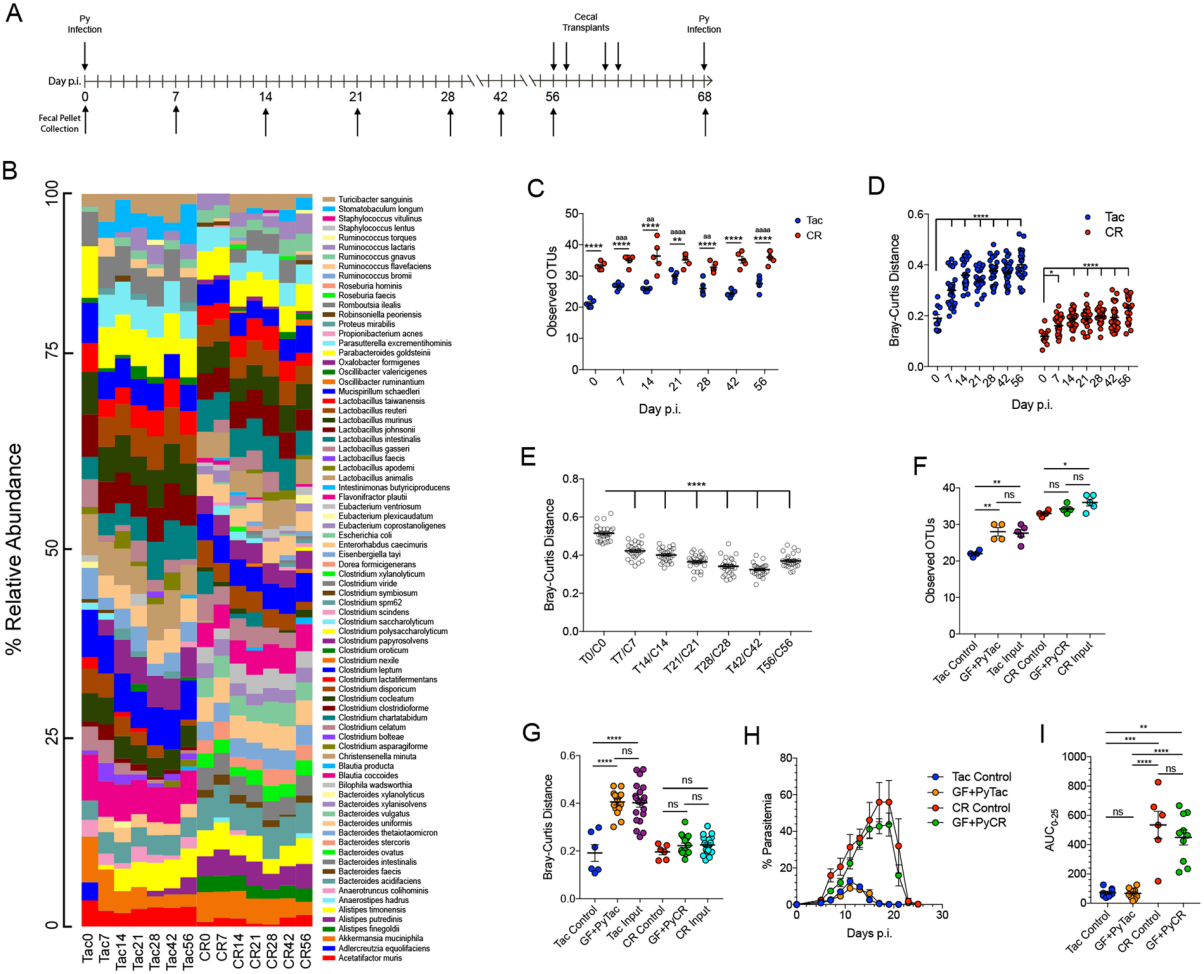
Gut bacterial community changes occurring post-Py infection do not change susceptibility to future Py infections. (**A**) Experimental design of time course and cecal transplants along with fecal pellet collection times. (**B**) Relative taxonomic abundance of bacterial species during Py infection. (**C**) Alpha diversity (sample richness) between Tac and CR mice during infection using observed OTUs. Data were analyzed with a repeated measures two-way ANOVA with Dunnett’s post-hoc multiple comparison test for comparisons to Day 0 p.i. and Sidak’s post-hoc multiple comparison test to compare Tac and CR diversity at each time point. (**D**) Beta diversity (sample dissimilarity) between Tac and CR mice during Py infection using the Bray-Curtis distance metric; each time point is compared to the respective Day 0 time point. Data were analyzed by one-way ANOVA with Dunnett’s post-hoc multiple comparison test. (**E**) Matched beta diversity comparisons between Tac and CR mice at each time point. Data were analyzed by one-way ANOVA with Dunnett’s post-hoc multiple comparison test. (**F**) Bacterial community alpha diversity from fecal pellets taken from mice receiving cecal contents. Data were analyzed by unpaired two-tailed t test. (**G**) Bacterial community beta diversity from fecal pellets taken from mice receiving cecal contents. The comparisons are Tac control vs Tac control, GF + PyTac vs Tac control, CR control vs CR control, and GF + PyCR vs CR control. Data were analyzed by unpaired two-tailed t test. (**H**) Parasitemia of GF mice gavaged with post-Py Tac or post-Py CR cecal contents along with controls. (**I**) AUC of the parasite burdens shown in panel (H). Data were analyzed by unpaired two-tailed t test. Data (mean ± SE) in panels (B–G) are from one experiment (4–5 mice per group); panels (H,I) are cumulative results of 2 experiments (4–5 mice/group/experiment). 1 symbol, p < 0.05; 2 symbols, p < 0.01; 3 symbols, p < 0.001; 4 symbols, p < 0.0001; ^ns^not significant. *Tac and CR comparisons; ^a^Tac comparisons with Day 0; ^b^CR comparisons with Day 0.

It has been shown previously that baseline gut microbiota composition in resistant and susceptible mice is sufficient to modulate the severity of infection^19^, but it has also been shown that the gut microbiota can experience *Plasmodium*-induced inflammation-related changes^20,21^. To initially determine how severity of infection impacts changes to bacterial community compositions over the course of infection, mice from Tac and CR were infected with Py and cecal contents were extracted for 16S rRNA (V6–V8) sequencing. While this was not a truly longitudinal analysis, it showed changes in both Tac and CR mice after infection, primarily after clearance of Py (Supplementary Fig. 3). To confirm these results in a longitudinal analysis and increase the taxonomic assignment depth, Tac and CR mice were infected with Py and fecal pellets were collected at days 0, 7, 14, 21, 28, 42, and 56 p.i. for analysis of gut bacteria (Fig. 3A). Extracted DNA from fecal pellets was subjected to bacterial community analysis using 16S rRNA gene sequencing based on a new technology called MVRSION (Multiple 16S Variable Region Species-level IdentificatiON) that simultaneously utilizes all 9 hypervariable regions^25^.

During Py infection, there are changes in the relative species abundance in both Tac and CR mice (Fig. 3B). For example, the Tac microbiota becomes more diverse as soon as 7 days p.i., with a significant increase in species diversity (alpha diversity) measured by observed OTUs (Fig. 3C). This increase is based on the appearance of different bacterial species such as *Stomatobaculum longum* that are not present at day 0 p.i. (Fig. 3B,C). Compared to the Tac microbiota, the CR microbiota is significantly more diverse before and during infection (Fig. 3C); however, the species diversity of the CR microbiota does not change from baseline (Fig. 3C). The beta diversity, or dissimilarity, of bacterial communities in both the Tac and CR mice increases significantly during infection compared to their respective baselines, with an earlier increase in Tac mice at day 7 (Fig. 3D). The increase in beta diversity illustrates the taxonomic changes that occur due to Py infection. Of note, Bray-Curtis dissimilarity values range from 0 (identical similarity) to 1.0 (complete dissimilarity). Thus, while the increases in beta diversity are significant, they are relatively modest in both Tac (mean; day 0 = 0.19 to day 56 = 0.40) and CR mice (mean; day 0 = 0.12 to day 56 = 0.23) with both Tac and CR communities, respectively, remaining relatively similar amongst themselves. These data demonstrate that Py infection alone, in contrast to the severity of infection, influences the observed changes in gut bacterial communities. Interestingly, the dissimilarity between the Tac and CR microbiota compositions decreases during infection (Fig. 3E), demonstrating a moderate convergence in the different microbiota compositions. Since different gut microbiota profiles have been shown to modulate susceptibility to Py infection^19^, these changes were investigated to determine how they impact susceptibility to future Py infections.

Mice infected with Py develop sterilizing immunity to Py after one infection, precluding the ability to directly reinfect these mice. Therefore, cecal contents were taken from Py-infected Tac and CR mice on days 56, 57, 60, and 61 p.i. and gavaged into germ-free (GF) mice (Fig. 3A). The recipient mice, along with non-gavaged control Tac and CR mice, were infected with Py seven days after the last cecal-content transplant and parasitemia was tracked.

To confirm the GF mice had been colonized properly, fecal samples from recipient mice were collected on the day of Py infection (Fig. 3A) for bacterial community analysis. The species diversity of both the GF + PyTac and GF + PyCR samples was similar to the input diversity, which means the GF mice were successfully colonized and their gut bacteria populations mirror the donor mice that had previously been infected with Py (Fig. 3F). More specifically, the Tac control samples had significantly lower species diversity than the input contents (Fig. 3F), confirming the observed changes during Py infection (Fig. 3C). Similarly, the Tac input and GF + PyTac samples are significantly dissimilar to the Tac controls but not each other (Fig. 3G). In the CR samples, the CR input samples had a slightly higher species diversity than the CR control (Fig. 3F), but this did not result in a bacteria community that was overall dissimilar (Fig. 3G). Following infection, the GF + PyTac and GF + PyCR contents phenocopied parasitemia in the respective Tac and CR control mice with regards to the infection kinetics (Fig. 3H) and overall parasite burden (Fig. 3I). These data suggest that Py-induced changes in gut bacterial communities do not change resistance or susceptibility to future Py infections.

### Cecal and Plasma Metabolite Profiles Vary Between Tac and CR Mice

One mechanism by which gut microbiota can influence host homeostasis is through the production of metabolites. Different metabolites can have various effects on the host: nucleotides in the gut can reduce inflammation, while tryptophan metabolites can activate the aryl hydrocarbon receptor (AhR) and lead to an anti-inflammatory and xenobiotic clearance response^26–28^. To this end, SI and cecal contents as well as plasma were extracted from mice at days 0, 7, 14, 21, 28, and 60 p.i. and characterized by mass spectrometry.

**Figure 4:**
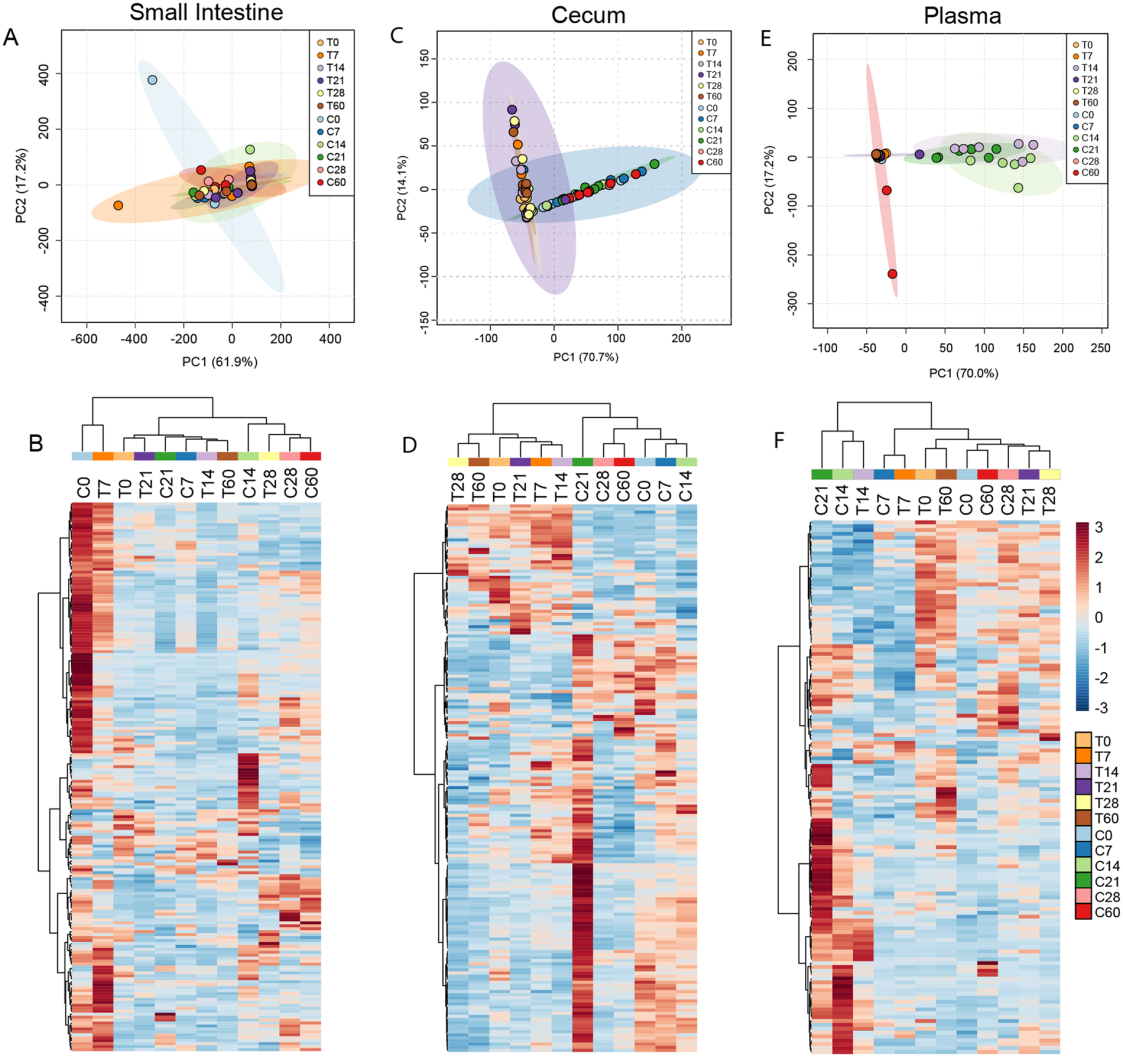
Metabolite profiles in selected tissues during Py infection. Principal Component Analysis (PCA) plot showing similarity of metabolite profiles in Tac and CR samples in the small intestine (**A**), cecum (**C**), and plasma (**E**). Ellipses contain the 90% confidence area of each group. Metabolite profile heatmap from the small intestine (**B**), cecum (**D**), and plasma (**F**). Sample group averages and metabolites are clustered using Ward’s method and Euclidean distance measure. Data are the cumulative results of 2 experiments (3 mice/group/experiment). Scale bar represents scaled relative abundance of metabolites.

In the SI, no distinct clustering between Tac and CR samples is observed, with the exception of two noted outliers, one Tac sample at day 7 p.i. and one CR sample at day 0 p.i. (Fig. 4A,B). In contrast, the cecal PCA plot shows distinct differences between Tac mice and CR mice (Fig. 4C). In the CR mice, a similar metabolite profile is seen at days 0, 7, and 14 p.i.; however, at day 21 p.i., which correlates to the peak of severe infection, the metabolites in the cecum become much more abundant (Fig. 4D). This enrichment decreases by day 28 p.i. to below-baseline levels for metabolites in the bottom-half of the heatmap and remain low even one month after clearance of the infection. In contrast to CR mice, cecal metabolites remain largely stable in Tac mice over the course of infection, with only a few metabolites decreasing at days 28 and 60 p.i. (Fig. 4D).

Tac and CR plasma metabolite profiles follow similar kinetics during infection. The most pronounced changes during infection are the similar shifts in metabolites at the peak of infection: day 14 p.i. in Tac mice and days 14 and 21 p.i. in CR mice (Fig. 4E). The clustering of these samples explains a robust 70% of the variation. The metabolite profiles seen in naïve mice become inverted, with abundant metabolites at day 0 p.i. becoming depleted, while less abundant metabolites becoming enriched at the peaks of infection in Tac and CR mice (Fig. 4F). In addition, after Py clearance, the metabolite profiles in both Tac and CR mice largely return back to pre-infection levels by day 60 p.i. (Fig. 4F).

Overall, Tac and CR mice have different metabolite profiles before and during infection, and the changes in cecal metabolite concentrations during infection correlates with the severity of infection. Meanwhile, the plasma metabolite dynamics appear to depend on the kinetics of infection more so than the severity of infection.

### Py Infection Causes Differential Liver Damage in Mice During Py Infection

**Figure 5:**
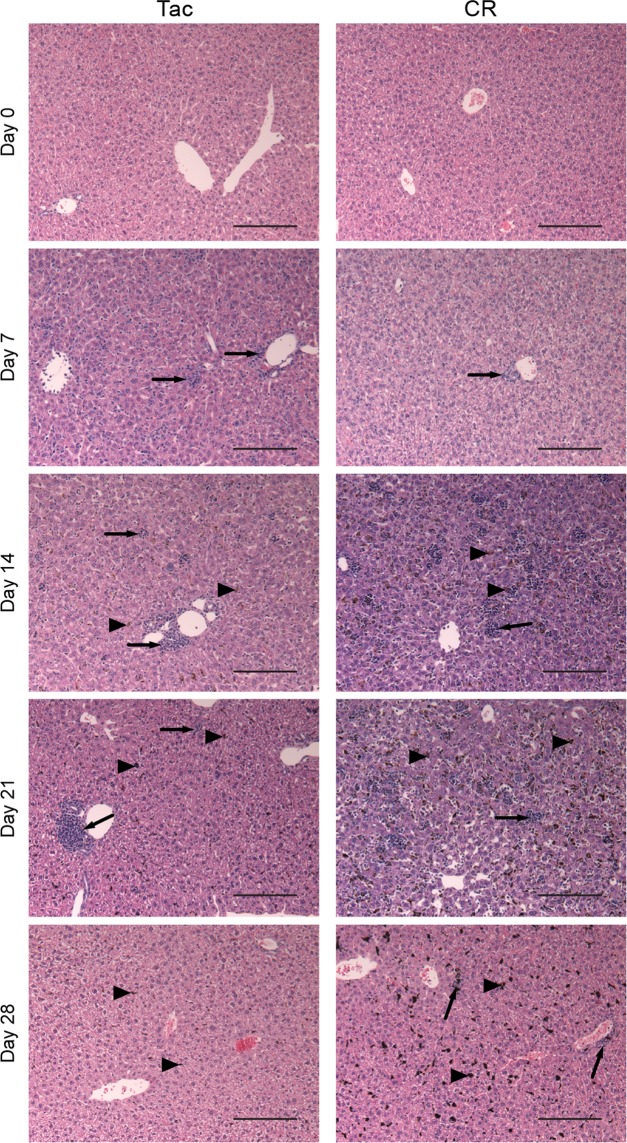
Severity of liver damage following Py infection correlates with both parasitemia burden and kinetics. Histology of representative livers (3 mice/group/time point) stained with H&E in naive and Py-infected mice. Scale bar = 100 µm; magnification = 20x. Arrowheads indicate hemozoin deposition; arrows indicate immune cell infiltration.

Differential cecal metabolites, noted gross anatomical changes in the liver of Py infected mice (data not shown), and the observation that *Plasmodium* infections cause fibrotic lesions in the liver^29^ led us to investigate a potential role of the liver in Py-induced changes in gut homeostasis. The gut-liver axis has been well-established^30–33^, and can influence metabolite profiles and microbial community structures through different mechanisms. Livers were extracted from mice at days 0, 7, 14, 21, and 28 days post-Py infection and stained with hematoxylin and eosin (H&E). Before infection, normal liver structure is observed in Tac and CR mice (Fig. 5). After Py infection, immune cells begin to infiltrate the liver and disruption of the liver architecture can be seen. In CR mice, the infiltrating cells remain in close proximity to the vasculature, while in Tac livers the infiltrating cells appear to invade further into the liver tissue (Fig. 5). At day 14, which is around peak parasitemia for Tac mice, immune cell infiltration increases and modest hemozoin deposition is seen (Fig. 5). In CR mice at day 14 p.i., the immune cell infiltration has progressed deeper into the tissue, and more extensive hemozoin deposition is seen compared to the Tac livers; the liver architecture also becomes further disrupted (Fig. 5). By day 21 p.i., Tac mice have cleared the infection; however, low levels of hemozoin and infiltrating immune cells are still present (Fig. 5). At day 28, the Tac liver architecture has returned to its pre-infection state and the infiltrating immune cells have left, but small amounts of hemozoin can still be seen within the tissue (Fig. 5). In comparison, day 21 p.i. is peak parasitemia for the CR mice, which display a more severe infection. Consistently, the liver architecture is highly disrupted, with infiltrating immune cells and hemozoin scattered throughout the tissue (Fig. 5). Liver architecture is partially restored by day 28 with only few infiltrating immune cells around the vasculature (Fig. 5). Compared to Tac livers at day 21 and day 28 p.i., there is notably more hemozoin in CR livers. Collectively, the severity of blood stage infection leads to much more pronounced and prolonged damage to the liver.

### Tac and CR Mice Have Different Bile Acid Profiles Before and During Py Infection

**Figure 6:**
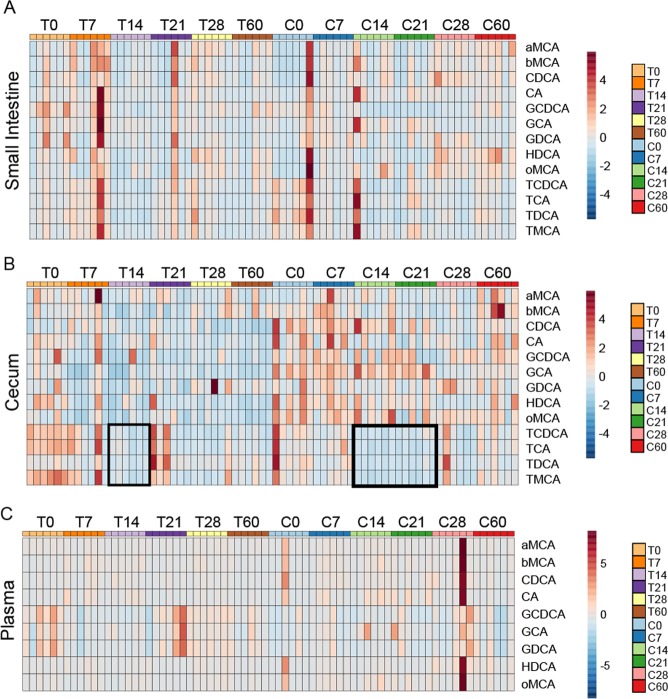
Severity of Py infection correlates with loss of specific cecal bile acids. (**A**) Small intestine bile acid heatmap. (**B**) Cecal bile acid heatmap. (**C**) Plasma bile acid heatmap. Data are cumulative over 2 experiments (3 mice/group/experiment). Scale bars indicate scaled relative abundance of bile acids. aMCA = alpha-muricholic acid; bMCA = beta-muricholic acid; CDCA = chenodeoxycholic acid; CA = cholic acid; GCDCA = glycochenodeoxycholic acid; GCA = glycocholic acid; GDCA = glycodeoxycholic acid; HDCA = hyodeoxycholic acid; oMCA = omega-muricholic acid; TCDCA = taurochenodeoxycholic acid; TCA = taurocholic acid; TDCA = taurodeoxycholic acid; TMCA = tauromuricholic acid.

One mechanism by which the liver can influence gut microbial communities is through bile acid production. Bile acids are detergent-like molecules that can be metabolized by gut bacteria, disrupt bacterial cell membranes, and act as signaling molecules to the intestinal epithelial cells^34–37^. We hypothesized that bile acid production would be altered as a function of the severity of infection. To test this hypothesis, bile acids were analyzed at days 0, 7, 14, 21, 28, and 60 p.i. in the SI, cecum, and plasma of Tac and CR mice. In the SI, the most notable pattern in bile acid expression is that the Tac mice have a significant decrease in almost half of the detected bile acids at day 14 compared to pre-infection bile acid levels (Fig. 6A, Supplementary Fig. 4). In terms of individual bile acids, conjugated bile acids glycochenodeoxycholic acid (GCDCA), taurochenodeoxycholic acid (TCDCA), and taurodeoxycholic acid (TDCA) in Tac and CR show significant changes at several time points (Supplementary Fig. 4).

In the cecum, CR mice tend to have higher relative bile acid concentration before and during infection compared to Tac mice (Fig. 6B, Supplementary Fig. 5). However, both Tac and CR mice exhibit a significant decrease in specific bile acids during infection, with the largest changes occurring in the taurine-conjugated bile acids. In particular, taurine-conjugated bile acids become depleted at day 14 in Tac mice with a mild recovery before a long-term depletion up to day 60 p.i. (Fig. 6B, Supplementary Fig. 5). CR mice have a similar depletion at day 14 that extends to day 21 p.i., consistent with the more severe infection in CR mice, but these bile acid levels return to baseline levels by clearance of the parasite at day 28 p.i. (Fig. 6B, Supplementary Fig. 5). Additionally, several bile acids are significantly higher in CR mice over the course of the infection compared to Tac, such as glycocholate (GCA) and omega-muricholate (oMCA) (Fig. 6B, Supplementary Fig. 5).

Plasma bile acids were low in abundance and were largely unchanged over the course of infection. The two noted exceptions were in Tac mice: glycine-conjugated bile acids GCDCA and glycodeoxycholate (GDCA) decrease at days 7 and 14 p.i. and day 7 p.i., respectively (Fig. 6C, Supplementary Fig. 6); alpha-muricholate (aMCA) significantly decreases at days 7 and 14 p.i. while beta-muricholate (bMCA) is significantly more abundant at those same time points (Supplementary Fig. 6).

Like the metabolomics data (Fig. 4), the cecal bile acid profiles in Tac and CR are different at baseline and change differentially due to mild or severe infection. In this case, however, the milder Py infection induces long-lasting depletion of bile acids.

### Predicted Functional Changes in the Microbiota Align with Combined Datasets

**Figure 7:**
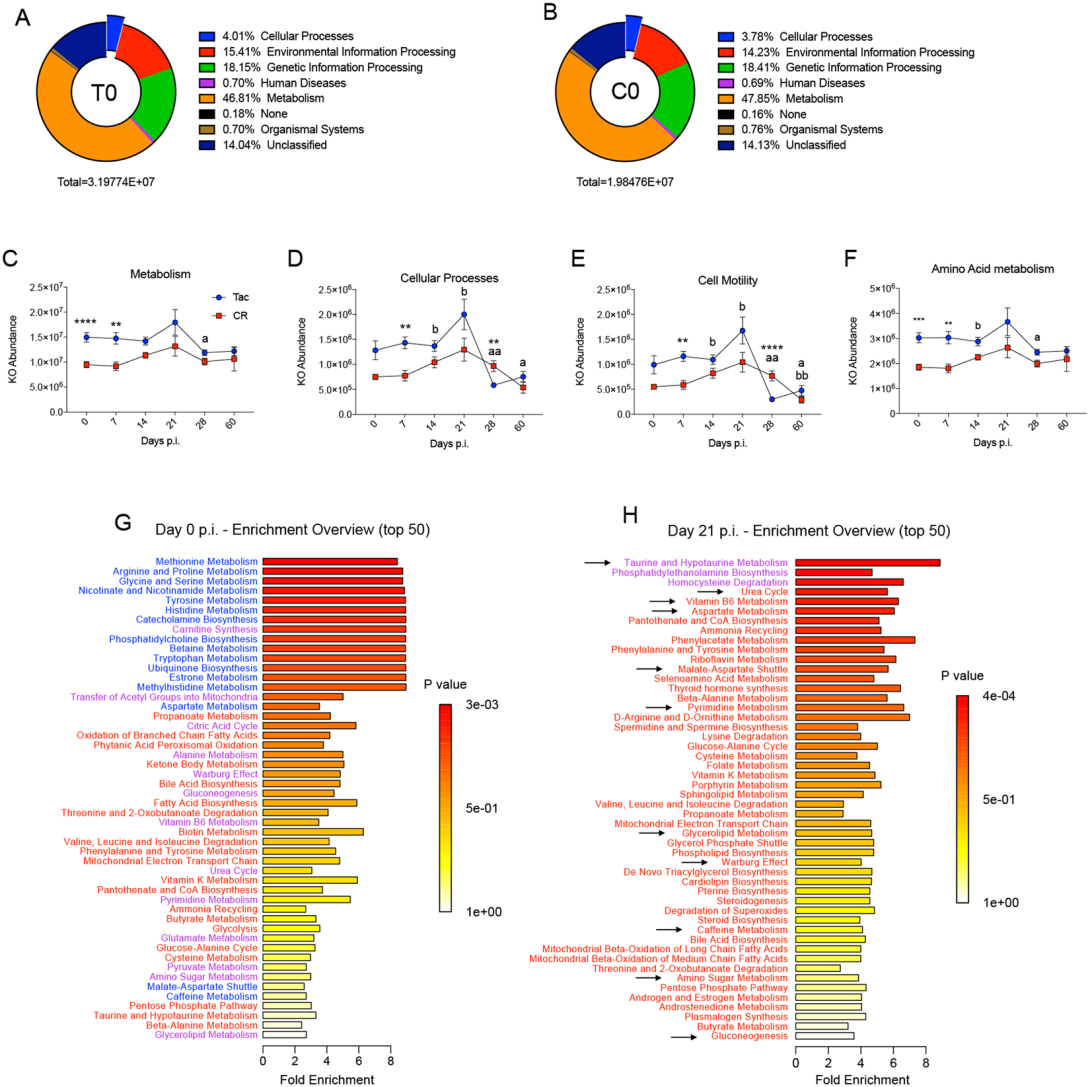
Severe Py infection increases the predicted functional capacity of the gut microbiota. (**A**) KO proportions and total abundance in the Tac cecal microbiota at day 0 p.i. (**B**) KO proportions and total abundance in the CR cecal microbiota at day 0 p.i. (**C**) KO abundances during Py infection classified to the level 1 KEGG category “Metabolism” between Tac and CR microbiota. (**D**) KO abundances during Py infection classified to the level 1 KEGG category “Cell Processes” between the Tac and CR microbiota. (**E**) KO abundances during Py infection classified to the level 2 KEGG category “Cell Motility” between the Tac and CR microbiota. (**F**) KO abundances of the “Amino Acid metabolism” category during Py infection. (**G**) Cecal metabolite pathway enrichment between Tac and CR mice at day 0 p.i. Blue means a pathway is enriched in Tac while red is enriched in CR; purple indicates enrichment in both. (**H**) Cecal metabolite pathway enrichment at day 21 p.i. Arrows represent pathways that were enriched in Tac at ay 0 p.i. but are enriched in CR at day 21 p.i. and vice versa. Data in (**C**–**F**) were analyzed by unpaired two-tailed t-test. (**G**,**H**) Data were analyzed using Metaboanalyst’s Enrichment Analysis based on the globaltest algorithm and are Holm-Bonferroni adjusted. Data (mean ± SE) originate from Supplementary Fig. 3 samples and are cumulative results (n = 2–3 mice/group/experiment) of two experiments. 1 symbol, p < 0.05; 2 symbols, p < 0.01; 3 symbols, p < 0.001; 4 symbols, p < 0.0001. *Tac and CR comparisons; ^a^Tac comparisons with Day 0; ^b^CR comparisons with Day 0.

We have observed many changes within the host and the microbiota during infection. To investigate how the observed taxonomic and metabolite profiles interact, PICRUSt was utilized to predict the functional capacity^38^ of cecal bacteria in Tac and CR mice based on the sequencing data in Supplementary Fig. 3. Once in PICRUSt, KEGG ortholog (KO; a characterized gene or protein within a network) abundances were assigned to each sample; KOs were then categorized according to the KO reference hierarchy and analyzed^39–41^. When naïve Tac and CR mouse KO abundances are compared, the proportional abundances of each category are similar, but in spite of the lower species diversity (Supplementary Fig. 3B) the naïve Tac microbiota have approximately 40% more KOs than the CR microbiota (Fig. 7A,B). “Metabolism” was the largest category, containing almost 47% of total KOs during infection. Tac microbiota possess significantly more KOs than CR mice at days 0 and 7 p.i., while from day 14 p.i. onward the Tac and CR microbiota possess similar KO abundances (Fig. 7C). The observed change in the functional capacity in the CR microbiota by day 14 p.i. precedes the large taxonomic changes and metabolomics changes observed at day 60 p.i. and day 21 p.i., respectively. Each level 1 KEGG category follows this same pattern except for one, “Cellular Processes”; Tac and CR microbiota are significantly different at day 7 p.i., but not at day 0, indicating that Py infection differentially affects the Tac and CR microbiota during early infection (Fig. 7D). Similarly, the CR microbiota undergoes significant functional changes in Cellular Processes at day 21 p.i. compared to the day 0 p.i. CR microbiota (Fig. 7D). The only level 2 subcategory under Cellular Processes that also changes significantly from day 0 to day 21 p.i. is “Cell Motility”, which includes flagellar assembly and bacterial chemotaxis (Fig. 7E). Finally, the Cellular Processes KOs at day 28 and day 60 p.i. correlate with the taxonomic changes: the Tac microbiota changes significantly at day 28 p.i., while the CR microbiota shows a delayed change at day 60 p.i., effectively “catching up” to the Tac microbial community structure. When comparing the PICRUSt data to the taxonomic data in Supplementary Fig. 3, the bloom in the S24-7 bacterial family (Supplementary Fig. 3A) correlates to a loss in the Cellular Processes functional capacity, and more specifically a loss in the microbiota’s capacity for cell motility (Fig. 7D,E). Taken together, naïve Tac and CR microbiota each have distinctly different functional profiles; after Py infection, the functional capacity of these two bacterial communities become indistinguishable, even after the parasite is cleared. In the case of some KO categories like Cellular Processes and Cell Motility, the KO abundances correlate closely with the taxonomic changes seen in Tac and CR microbiota during convalescence.

The metabolomics data can be used to validate the predicted functionality of the cecal microbiotas. Tac mice are predicted to have a significantly higher functional capacity for “Amino Acid metabolism” (Fig. 7F) compared to CR mice. When compared to the observed metabolite abundances, the top enriched pathways at day 0 p.i. are related to metabolism of various amino acids, and this enrichment is associated with the Tac phenotype (Fig. 7G). In contrast, at day 21 p.i., the enriched pathways have changed and the majority are now associated with the CR phenotype (Fig. 7H); this shift correlates to the large increase in metabolite abundances in the CR cecum at day 21 p.i. (Fig. 4D). Overall, the functional predictions in the ceca of Tac and CR mice fit well with the taxonomic and metabolomic data presented.

## Discussion

**Figure 8:**
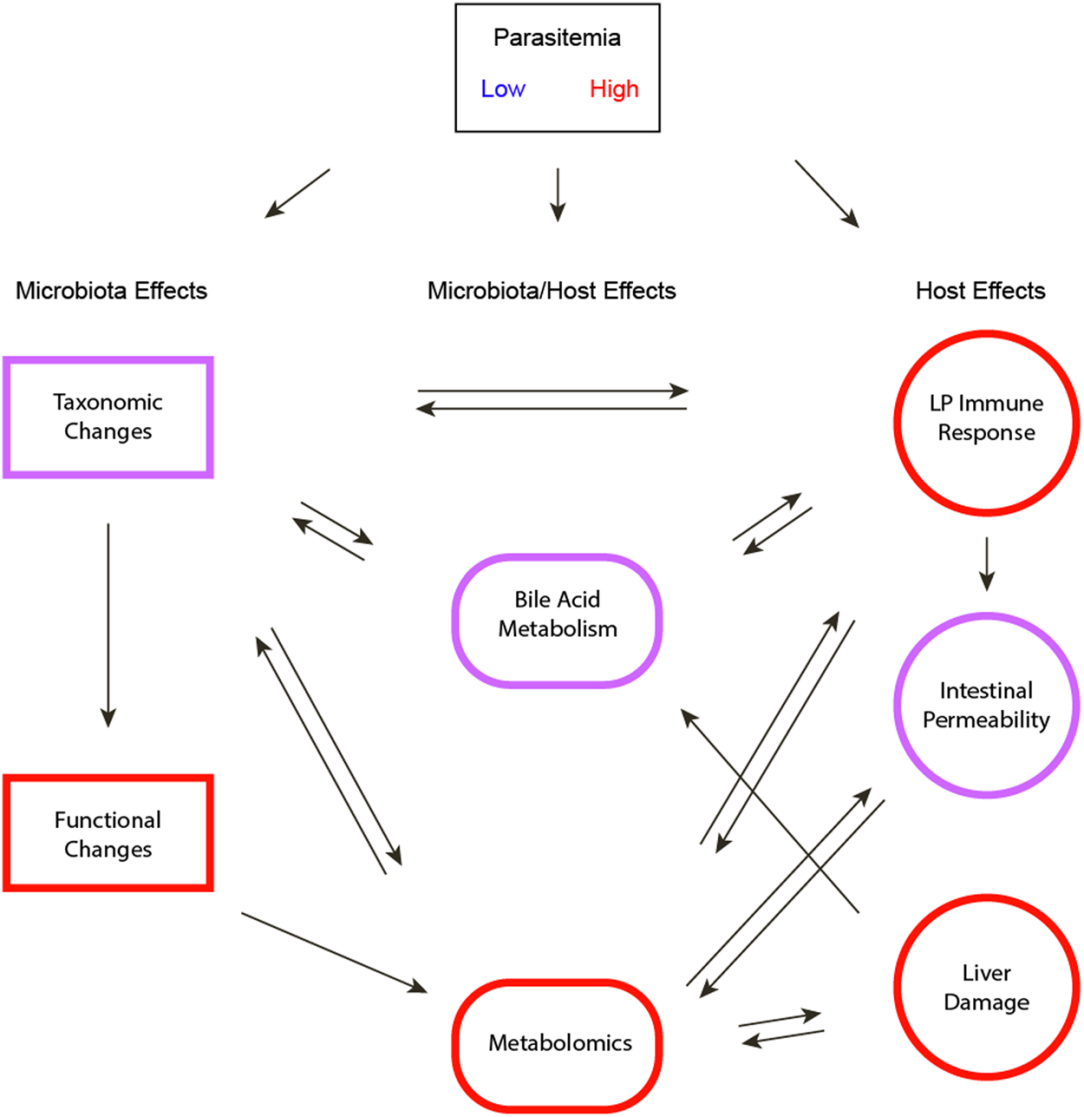
Schematic of the different host and microbiota factors. Arrows represent the ability of one factor to influence another. Colors represent differential effects driven by low or high parasitemia.

In this study, we have shown that gut homeostasis is differentially disrupted by severe malaria compared to mild malaria. We examined several factors of gut homeostasis, including intestinal permeability, the intestinal immune system, gut microbiota and metabolites, and the gut-liver axis. Altogether, severe malaria differentially influences gut homeostasis with distinct actions (Fig. 8). During infection, the LP in CR mice with severe malaria has a larger influx of CD8 T cells, monocytes, neutrophils, and TCRgd cells, all of which can produce proinflammatory cytokines. The microbiota of both Tac and CR mice differentially change during infection, with larger changes occurring earlier in Tac mice. The taxonomic changes in Tac and CR mice lead to enrichment of different bacterial species, highlighting the differential modulation of microbiota by Py infection. Initially, CR microbiota have a sparse predicted functional profile compared to Tac microbiota; during infection, the CR and Tac predicted functional capacity becomes more similar, reflecting the taxonomic similarity at the family level post-Py clearance. The cecal metabolite profiles in CR mice reflect the parasite burden, with the peak of infection mirroring a large increase in metabolite abundances, while Tac mice do not show a similar increase. CR mice also show major liver damage during infection with more extensive damage and hemozoin deposition compared to asymptomatic Tac mice. Finally, cecal bile acid profiles in Tac mice show a prolonged depletion during infection compared to CR mice, with CR bile acid abundances generally returning to baseline levels by days 28 and 60 p.i. Taken together, these data show patterns of gut homeostasis disruption during severe malaria.

While previous studies have shown changes to gut microbiota during *Plasmodium* infection as well, our data is unique in that it shows that there are long-term changes that occur during infection that persist after the infection is cleared. Mooney *et al*. showed that during *Plasmodium yoelii nigeriensis* infection, there were shifts in murine gut microbiota, but these returned to baseline within 30 days p.i. and were taxonomically restricted primarily to the phylum level with only a few noted changes at the genus level^21^. Taniguchi *et al*. also showed shifts in the gut microbiota of C57BL/6 mice infected with *P. berghei* ANKA, which models cerebral malaria, but not BALB/c mice, which do not model cerebral malaria^20^. Of note, all the *P. berghei* ANKA infected C57BL/6 mice succumbed to cerebral malaria, so it is unknown if these changes affect parasite burden, immunity to malaria, or would remain after clearance of infection. We have extended these analyses to show that the gut microbiota undergoes long-term, persistent changes down to the species level due to Py infection and that these changes do not affect susceptibility to future Py infections.

We have also looked more closely at the LP immune response during Py infection. Previously, it had been observed that during *P. yoelii nigeriensis* infection, mononuclear cells infiltrate the LP up to 10 days p.i., with a large portion of these identified as inflammatory monocytes (Ly6C^+^ Ly6G^−^)^21^. Using Py 17XNL instead of *P. yoelii nigeriensis*, we have also found that large numbers of monocytes infiltrate into the LP of mice with high parasitemia 21 days p.i. before returning to baseline, while mice with low parasitemia show a significant increase 60 days p.i. In addition, we have observed a significant increase in macrophage infiltration at 7 days p.i. and 60 days p.i. We have corroborated the previously observed early mononuclear cell infiltration and extended the analysis further p.i. and to more cell types.

A potential mechanism for how severe malaria differentially impacts gut homeostasis may be due to parasite sequestration in the gut vasculature. In severe malarial anemia in humans, for instance, the bulk of parasite sequestration occurs in the vasculature of the small and large intestines; patients with cerebral malaria also show a large amount of parasite sequestration in the gut along with the brain^42^. Sequestration in the vasculature could lead to inflammation which in turn would damage the gut tissue, disrupting tissue homeostasis.

One promising avenue for further investigation within the malaria-gut microbiota axis involves bile acid metabolism in the gut. In the intestine, bile acids aid in digestion but can also behave as signaling molecules or bacteriostatic agents. As detergent-like molecules, bile acids can weaken or destroy bacterial cell membranes, keeping bacterial growth in check^37,43^. In both Tac and CR mice, the initial liver damage and depletion of bile acids correlates with the increase in gut bacterial diversity during and after infection, pointing to the potential role of bile acids in shaping the gut microbiota before and during Py infection.

Signaling by bile acids in the intestine can occur through the G protein-coupled receptor TGR5. CDCA and lithocholic acid (LCA)/DCA are the strongest ligands, with other bile acids binding with lesser affinity; conjugation with taurine or glycine makes binding and activation more effective^34–36^. TGR5 is found in the intestine as well as in extra-intestinal tissues such as lung, spleen, and bone marrow^44^. Since plasma levels of bile acids did not vary between Tac and CR mice during infection, it is unlikely that activation of TGR5 in extra-intestinal tissue is involved. However, TGR5 activation on intraepithelial lymphocytes leads to the expression of GLP-1 (glucagon-like peptide 1) which in turn inhibits proinflammatory cytokine production^45^. As Tac mice have a greater abundance of taurine-conjugated bile acids than CR mice initially, TGR5 signaling may be play a protective anti-inflammatory role early in Py infection.

While there is still much work to be done, these results identify the complex network of interactions that influence gut homeostasis during Py infection in mice and provide an extensive characterization of how different factors in gut homeostasis respond during mild versus severe Py infection. Many of the perturbations in gut homeostasis were associated with a more severe infection. Given that many parasitized red blood cells sequester in intestinal villi in humans, it is paramount that future work begin assessing the effect of *Plasmodium* infections on human gut homeostasis.

## Materials and Methods

### Animals and Housing

Female C57BL/6 mice 6–8 weeks old were purchased from Taconic Biosciences (Hudson, NY) and Charles River Laboratories (Wilmington, MD). Germ-free (GF) mice were purchased from Taconic Biosciences. All mice were housed in a specific pathogen-free facility and acclimatized for a minimum of 7 days before starting experiments. Animals were fed the NIH-31 diet (Modified Open Formula Mouse/Rat Irradiated Diet; Harlan 7913; Envigo, Indianapolis, IN) and provided autoclaved, non-acidified municipal water ad libitum. The mice were kept on a 12-hour light/dark cycle from 6 AM to 6 PM and 6 PM to 6 AM, respectively. All animal handling and experimentation were reviewed and approved by the University of Louisville Institutional Animal Care and Use Committee based on the recommendations of the Guide for the Care and Use of Laboratory Animals of the National Institutes of Health. All experiments were performed in accordance with these relevant guidelines and regulations.

### *Plasmodium* Infection and Evaluation of Parasitemia

Mice were infected with *Plasmodium yoelii* 17XNL by intravenous injection of 1 × 10^5^ infected red blood cells (RBCs) in 200 uL of saline prepared from frozen stock. Parasitemia (i.e. percentage of total infected RBCs) was evaluated by flow cytometry between days 5–30 post-infection (p.i.) via blood taken from the tails of infected mice. Approximately 5 µL of whole blood was diluted in 100 µL of PBS followed by fixation in 0.00625% glutaraldehyde. The samples were then stained: CD45.2-APC (clone 104; Biolegend, San Diego, CA), Ter119-APC/Cy7 (clone TER-119; Biolegend, San Diego, CA), dihydroethidium (MilliporeSigma, St. Louis, MO), and Hoechst 33342 (MilliporeSigma, St. Louis, MO). After staining, samples were resuspended in flow cytometry buffer and analyzed; RBCs were gated by Ter119^+^CD45.2^−^ followed by gating the infected subpopulation on dihydroethidium^+^Hoechst 33342^+^ to find the percentage of infected RBCs.

### GF Cecal Content Transplant

Tac and CR cecal donor mice were infected with Py; at day 56 p.i., GF mice were received. GF mice were colonized immediately upon arrival with cecal contents from either the infected Tac or CR donor mice. GF mice were gavaged daily with cecal contents for a total of 4 treatments and then rested for 1 week before Py infection. Fecal pellets were collected after gavage to ensure colonization recapitulation of donor microbiota.

### Liver Histology

Liver samples were collected from mice at days 0, 7, 14, 21, and 28 p.i. Livers were extracted from mice, trimmed of connective tissue, and placed into Tissue-Tek Uni-Cassettes (Sakura Finetek, Torrance, CA) in 10% neutral buffered formalin (MilliporeSigma, St. Louis, MO) for fixation. Livers were then processed using a graded ethanol series and embedded in paraffin. The paraffin sections were cut into 5 μM-thick slices using a microtome and stained with hematoxylin and eosin (H&E). All stained sections were examined by light microscopy using an Olympus BX41 microscope. Representative images are shown (magnification −20X).

### Intestinal Permeability Assay

Intestinal permeability was measured using 4 kD FITC-dextran (MilliporeSigma, St. Louis, MO). Mice were fasted for 4 hours followed by oral gavage of ~42 mg FITC-dextran/100 mg of body weight in 200 µL of PBS, or 8.4 mg/200 µL/mouse. Three hours post-gavage, serum was collected, diluted 1:1 in PBS to reach 100 µL final volume, and read on a spectrophotometric plate reader for fluorescence intensity (Excitation at 485 nm and emission at 528 nm).

### Lamina Propria Immune Cell Analysis

Lamina propria (LP) immune cells were isolated at days 0, 7, 14, 21, 28, and 60 p.i. using the mouse Lamina Propria Dissociation Kit (Miltenyi Biotec, Auburn, CA) according to the manufacturer’s instructions. Briefly, small and large intestines were extracted from mice and cut open longitudinally and laterally into approximately 0.5 cm long pieces. The samples were then incubated and washed to dissociate the epithelial layer. The resulting samples were then run on a gentleMACS dissociator (Miltenyi Biotec, Auburn, CA) and filtered to obtain a single-cell suspension. Samples were split and either only surface stained or surface stained and intracellularly stained with fluorescence-conjugated antibodies. Antibodies were resuspended in FACS buffer (1x PBS, 0.02% sodium azide, and 1% FCS) with FC block (CD16/32 clone 2.4G2) for surface staining for 15 minutes at 4 °C followed by fixation with Fixation Buffer (Biolegend, San Diego, CA), while intracellular staining was carried out with the eBioscience Foxp3/Transcription Factor Staining Buffer Set (ThermoFisher, Waltham, MA) according to the manufacturer’s instructions. Samples were collected on a BD LSRFortessa (BD Biosciences, San Jose, CA) and analyzed using FlowJo software for Mac, version 10.4.2 (FlowJo, Ashland, OR).

### Antibodies

CD3-FITC clone 145-2C11, CD4-APC-Cy7 clone RM4-5, CD8-FITC clone 53-6.7, CD45-AF700 clone 104, CD11b-PE-Cy7 clone M1/70, CD11c-BV450 clone N418, CD19-PerCP-Cy5.5 clone 6D5, CD49b-APC clone DX5, F4/80-BV421 clone BM8, IL-17A-PE-Cy7 clone TC11-18H10.1, Ly6G-APC clone 1A8, and TCRγδ-BV421 clone GL3, and were purchased from Biolegend (San Diego, CA). Ly6C-PerCP-Cy5.5 clone AL-21, RORγt-BV650 clone Q31-378, and Siglec F-PE clone E50-2440 were purchased from BD Biosciences (San Jose, CA). Foxp3-PE clone 150D/E4 was purchased from ThermoFisher Scientific (Waltham, MA).

### Metabolite Screening and Bile Acid Analysis

Ceca, small intestine, and plasma samples were collected from Tac and CR mice at days 0, 7, 14, 21, 28, and 60 p.i. The ceca and small intestines were flushed with extraction buffer (a mix of 40:40:20 HPLC grade methanol, acetonitrile, and water with 0.1% formic acid overall), flash frozen in liquid nitrogen, and stored at −80 °C. Samples were shipped overnight on dry ice to our collaborators for an untargeted metabolomics screen and targeted bile acid analysis. Sample preparation was the same for both mass spectrometric analyses. Shipped samples were extracted, dried, resuspended and immediately placed in a 4 °C mass spectrometer autosampler according to a method previously described^19^. 10 μL of sample was injected into the Ultra Performance Liquid Chromatography-High Resolution Mass Spectrometer (UPLC-HRMS), a Dionex Ultimate 3000 coupled to an Exactive Plus orbitrap mass spectrometer (ThermoFisher, Walham, MA, USA).

The untargeted metabolomic screen achieved separations using a Synergy Hydro-RP column (100 mm × 2 mm, 2.5 μm particle size, Phenomenex, Torrance, CA) at a flow rate of 200 μL/min. The mobile phase consisted of 97:3 HPLC grade water:methanol, 11 mM tributylamine, and 15 mM acetic acid labeled as solvent A, as well as HPLC grade methanol, labeled as solvent B. The mobile phase gradient was programmed accordingly: From 0 to 5 min, 0% B; from 5 to 13 min, 20% B; from 13 to 15.5 min, 55% B, from 15.5 to 19 min, 95% B; and from 19 to 25 min, 0% B, Eluent from the column was introduced into the mass spectrometer, an Exactive Plus orbitrap (ThermoFisher, Waltham, MA, USA) via an electrospray ionization (ESI) source set to negative mode. Instrument settings include: spray voltage of 3 kV, nitrogen sheath gas flow rate of 10 units, capillary temperature set at 320 °C, and an AGC target set to 3e6. Samples were analyzed at a resolution of 140,000 in full scan mode. The scan window included 85 to 800 *m/z* units from 0 to 9 min and 110 to 1000 *m/z* units from 9 to 25 min. Bile acids were analyzed by the same UPLC-HRMS instrument and column that was used as in the metabolomics analysis. The column compartment was kept at 40 °C and the flow rate was kept at 300 μL/min. Mobile phase composition was 0.1% formic acid in water labeled as solvent A and 0.1% formic acid in acetonitrile. The mobile phase gradient consisted of: 0% to 100% B from 0 to 13 min, 100% B from 13 to 14 min, 100% to 0% B from 14 to 14.5 min, 0% B from 14.5 min to 20.5 min. Eluent from this method was introduced into the mass spectrometer via a heated electrospray ionization (HESI) source also set to negative mode. Instrument settings include: spray voltage of 4.2 kV, nitrogen sheath gas flow of 25 units, capillary temperature set at 300 °C, and an AGC set to 3e6. Samples were analyzed at a resolution of 140,000 in full scan mode. The scan window was from 150–1000 *m/z* units.

The collected data for each tissue was normalized by tissue weight (small intestine and ceca) or volume (plasma) followed by median normalization. Tissue data was then formatted for and analyzed with MetaboAnalyst v4.0 (http://www.metaboanalyst.ca/MetaboAnalyst/faces/home.xhtml), an online tool for metabolomic analysis^46–49^. Using the Statistical Analysis tool, PCA plots and heatmaps were generated for the untargeted metabolomics screen; for the heatmaps, time point groups were collapsed using group averages, and Ward’s method was used for the clustering algorithm along with a Euclidean distance measure; relative abundance data was autoscaled to account for metabolites with very low or very high abundances. For bile acid heatmaps, neither the samples or features were clustered. The Enrichment Analysis tool was used to identify enriched pathways between Tac and CR mouse cecal samples at day 0 p.i. The library for analysis was the Pathway-associated Metabolite Sets and only metabolite sets containing at least 2 compounds were used. The tissue-normalized data for the untargeted metabolomics and bile acid screen in the SI, cecum, and plasma are available as Supplementary Tables 1–3 and 4–6, respectively.

### Gut Microbiota Analysis

Mouse ceca and fecal pellets were extracted and flash frozen in liquid nitrogen followed by storage at −80 °C. DNA was extracted using the QIAamp PowerFecal DNA kit (QIAGEN, Germantown, MD) according to the manufacturer’s instructions. DNA samples were then shipped overnight on ice packs to either the Integrated Microbiome Resource within the Centre for Comparative Genomics and Evolutionary Bioinformatics at Dalhousie University (IMR-CGEB, Halifax, NS, Canada) or the Genome Technology Access Center at Washington University (GTAC, St. Louis, MO) for sequencing.

Samples submitted to the IMR-CGEB were amplified using primers targeting the V6–V8 hypervariable regions. Sequencing was done on an Illumina MiSeq with 300 bp paired-end reads. Sequence analysis was done using the Microbiome Helper pipeline, which provides wrapper scripts for common bioinformatics tools^50^. Briefly, sequences were inspected with FastQC v0.11.5; the paired-end reads were stitched together with PEAR v0.9.6 and filtered with BBMap v37.24 with a quality score cutoff of 30^51–53^. The filtered FASTQ files were converted to FASTA using the FASTX toolkit v0.0.13.2 and chimeras were removed with VSEARCH v1.11.1^54,55^. QIIME v1.9.1 was then run for open reference OTU picking using SortMeRNA v2.0 and SUMACLUST v1.0.20 for reference picking and *de novo* OTU picking; alignment was done using PyNAST v1.2.2 and Greengenes 13_8^56-60^. The resulting OTU table was cumulative sum scaled (CSS). Analyses were done within QIIME to produce taxa plots as well as calculate alpha diversity and Bray-Curtis beta diversity. The map file containing sample metadata for analyses is included as Supplementary Table 7.

Samples submitted to the GTAC were amplified using a novel approach called MVRSION (Multiple 16S Variable Region Species-level IdentificatiON) that consists of 12 primer pairs that span portions of all 9 of the 16S rRNA hypervariable regions^25^. Upon receiving the OTU table from GTAC, it was CSS normalized and analyzed within QIIME to produce taxa plots and calculate alpha diversity and Bray-Curtis beta diversity. Both the map file and OTU table used for analyses are included as Supplementary Tables 8 and 9, respectively.

PICRUSt was used to predict the functional capacity of the samples sent to the IMR-CGEB^38^. The OTU table was filtered to remove *de novo* OTUs to produce a compatible OTU table. After filtering, the OTU table was uploaded to the Langille Galaxy server (http://galaxy.morganlangille.com/) running PICRUSt v1.1.1 for normalization and analysis. All analyses used KEGG Orthologs for functional predictions.

The joint analysis of the PICRUSt data and metabolite data utilized MetaboAnalyst’s Network Explorer tool. The KOs and metabolites were added as lists without fold changes for the day 0 time points in Tac and CR mice. The mode of analysis used the KEGG Global Metabolic Network and the table containing the significantly enriched pathways was downloaded.

The metadata map files and GTAC OTU table are included as Supplementary Tables 7–9.

### Statistical Analysis

Statistical analyses were performed using GraphPad Prism 7 software (GraphPad Software, La Jolla, CA, USA); the alpha value for each analysis was set at 0.05. Specific analyses are described in figure legends. For area under the curve (AUC) parasite burden analyses, the trapezoidal rule was used:

$${\rm{A}}{\rm{U}}{{\rm{C}}}_{({\rm{t}}1-{\rm{t}}-\mathrm{last})}=={\rm{\Sigma }}\,({{\rm{p}}}_{{\rm{i}}}++{{\rm{p}}}_{{\rm{i}}+1})\ast ({{\rm{t}}}_{{\rm{i}}+1}-{{\rm{t}}}_{{\rm{i}}})/2$$

where “p” is percent parasitemia at the designated time point “t”^61^.

## Supplementary information

Supplementary Information

Supplementary Table 1

Supplementary Table 2

Supplementary Table 3

Supplementary Table 4

Supplementary Table 5

Supplementary Table 6

Supplementary Table 7

Supplementary Table 8

Supplementary Table 9

## Data Availability

All data generated or analyzed during this study are included in this published article and the Supplementary information files. 16S rRNA gene sequences have been deposited in the NCBI Sequence Read Archive under the BioProject accession PRJNA489587 located at https://www.ncbi.nlm.nih.gov/bioproject/PRJNA489587/. The mapping files for both 16S rRNA sequencing experiments, the OTU table from GTAC, and the metabolomics data files are included as Supplementary data files with this manuscript.
