# Supplementary material for: Differential Sensitivity to Plasmodium yoelii Infection in C57BL/6 Mice Impacts Gut-Liver Axis Homeostasis
Source: Sci Rep. 2019 Mar 5;9:3472. doi: 10.1038/s41598-019-40266-6 (PMC6401097; doi:10.1038/s41598-019-40266-6)

Differential Sensitivity to *Plasmodium yoelii* Infection in C57BL/6 Mice Impacts Gut-Liver Axis Homeostasis

Joshua E. Denny[1], Joshua B. Powers[2], Hector F. Castro[2], Jingwen Zhang[3], Swati Joshi-Barve[3], Shawn R. Campagna[2], and Nathan W. Schmidt[1*]

Supplementary Information:

Figure Legends

Supplementary Figure 1. Gating strategies from the LP of mouse SI and LI. **A.** Gating strategy of single cell suspension and CD45+ cells. **B.** Gating strategy for CD4+ and CD8+ T cells. **C.** Gating strategy for TCRgd T cells and IL17+ TCRgd T cells. **D.** Gating strategy for Tregs, Th17 T cells, and IL17+ Th17 T cells. **E.** Gating strategy for neutrophils, monocytes, and macrophages.

Supplementary Figure 2. Large intestine lamina propria immune system changes during Py infection. Total cell numbers of **A.** CD45+ cells, **B.** CD8+ T cells, **C.** CD4+ T cells, **D.** Tregs, **E.** Gamma delta T cells (TCRgd), **F.** IL17+ TCRgd **G.** Th17 cells, **H.** IL17+ Th17 cells, **I.** Macrophages, **J.** Monocytes, and **K.** Neutrophils. Each time point was compared by one-way ANOVA with Tukey's post-hoc multiple comparison test. Data (mean±SE) are cumulative results of 2 experiments (3 mice/group/experiment). 1 symbol, $p < 0.05$; 2 symbols, $p < 0.01$; 3 symbols, $p < 0.001$; 4 symbols, $p < 0.0001$. * = Tac and CR LI comparisons; a = Tac LI comparisons to Day 0; b = CR LI comparisons to Day 0.

Supplementary Figure 3. The composition of gut bacteria populations changes after clearance of Py. **A.** Relative taxonomic abundance of bacterial families during Py infection. c = class, o = order. **B.** Alpha diversity (sample richness) between Tac and CR mice during infection using the Chao1 metric. **C.** Beta diversity (sample dissimilarity) between Tac and CR mice during Py infection using the Bray-Curtis distance metric; each time point is compared to the respective Day 0 time point. Data in **B-C** were analyzed by one-way ANOVA with Dunnett's post-hoc multiple comparison test. Data (mean±SE) are cumulative results of 2 experiments (2-3 mice/group/experiment). *p < 0.05; **p < 0.01; ***p < 0.001; ****p < 0.0001; ns= not significant.

Supplementary Figure 4. Small intestine bile acid profiles over Py infection. aMCA = alpha-muricholic acid; bMCA = beta-muricholic acid; CDCA = chenodeoxycholic acid; CA = cholic acid; GCDCA = glycochenodeoxycholic acid; GCA = glycocholic acid; GDCA = glycodeoxycholic acid; HDCA = hyodeoxycholic acid; oMCA = omega-muricholic acid; TCDCA = taurochenodeoxycholic acid; TCA = taurocholic acid; TDCA = taurodeoxycholic acid; TMCA = tauromuricholic acid. Data were analyzed by unpaired t-test. Data (mean±SE) are cumulative results (n=3 mice/group/experiment) of two experiments. 1 symbol, p < 0.05; 2 symbols, p < 0.01; 3 symbols, p < 0.001; 4 symbols, p < 0.0001. * = Tac and CR comparisons; a = Tac comparisons with Day 0; b = CR comparisons with Day 0.

Supplementary Figure 5. Cecal bile acid profiles over Py infection. aMCA = alpha-muricholic acid; bMCA = beta-muricholic acid; CDCA = chenodeoxycholic acid; CA = cholic acid; GCDCA = glycochenodeoxycholic acid; GCA = glycocholic acid; GDCA = glycodeoxycholic acid; HDCA = hyodeoxycholic acid; oMCA = omega-muricholic acid; TCDCA = taurochenodeoxycholic acid; TCA = taurocholic acid; TDCA = taurodeoxycholic acid; TMCA = tauromuricholic acid. Data were analyzed by unpaired t-test. Data (mean±SE) are cumulative results (n=3 mice/group/experiment) of two experiments. 1 symbol, $p < 0.05$; 2 symbols, $p < 0.01$; 3 symbols, $p < 0.001$; 4 symbols, $p < 0.0001$. * = Tac and CR comparisons; a = Tac comparisons with Day 0; b = CR comparisons with Day 0.

Supplementary Figure 6. Plasma bile acid profiles over Py infection. aMCA = alpha-muricholic acid; bMCA = beta-muricholic acid; CDCA = chenodeoxycholic acid; CA = cholic acid; GCDCA = glycochenodeoxycholic acid; GCA = glycocholic acid; GDCA = glycodeoxycholic acid; HDCA = hyodeoxycholic acid; oMCA = omega-muricholic acid. Data were analyzed by unpaired t-test. Data (mean±SE) are cumulative results (n=3 mice/group/experiment) of two experiments. 1 symbol, $p < 0.05$; 2 symbols, $p < 0.01$; 3 symbols, $p < 0.001$; 4 symbols, $p < 0.0001$. * = Tac and CR comparisons; a = Tac comparisons with Day 0; b = CR comparisons with Day 0.

Supplementary Figure 1

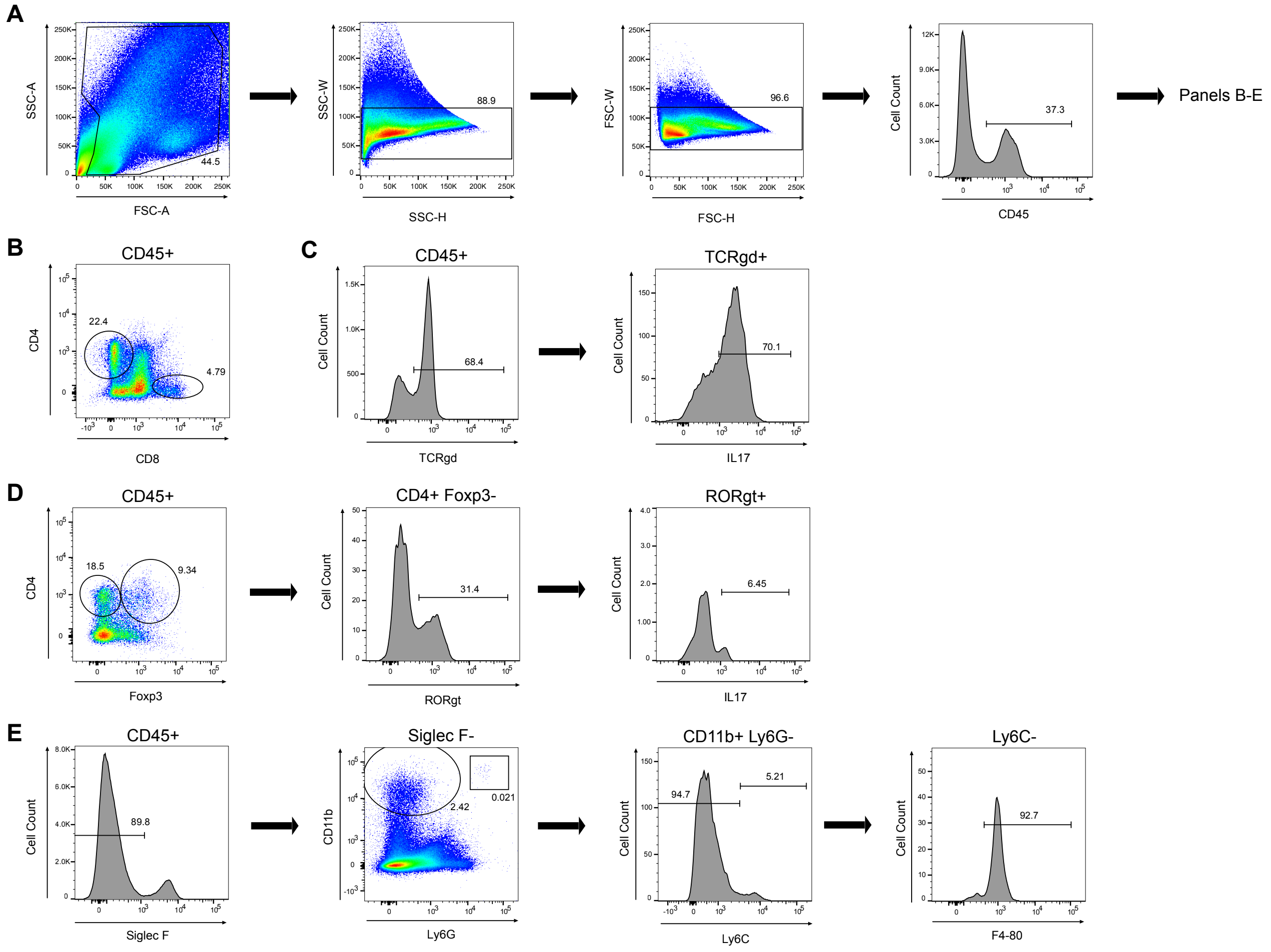

Supplementary Figure 2

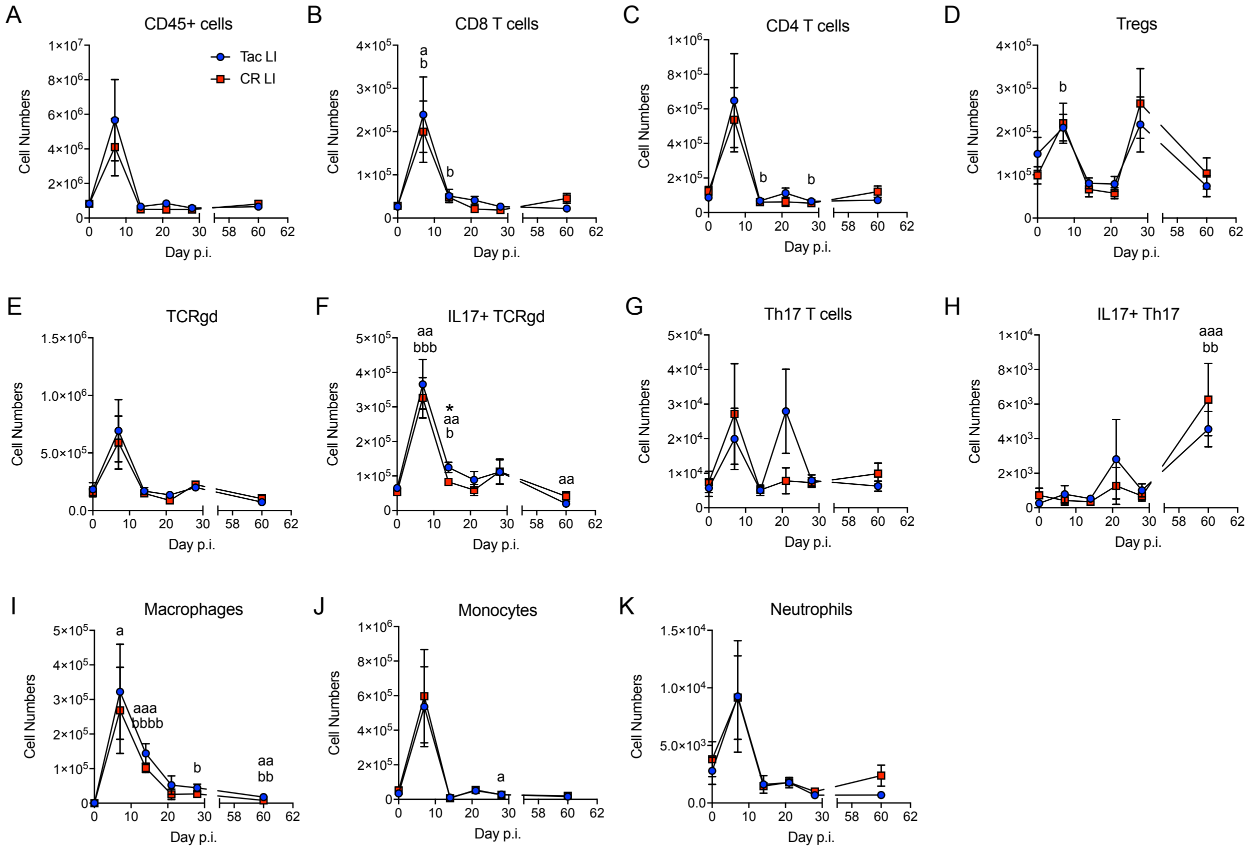

Supplementary Figure 3

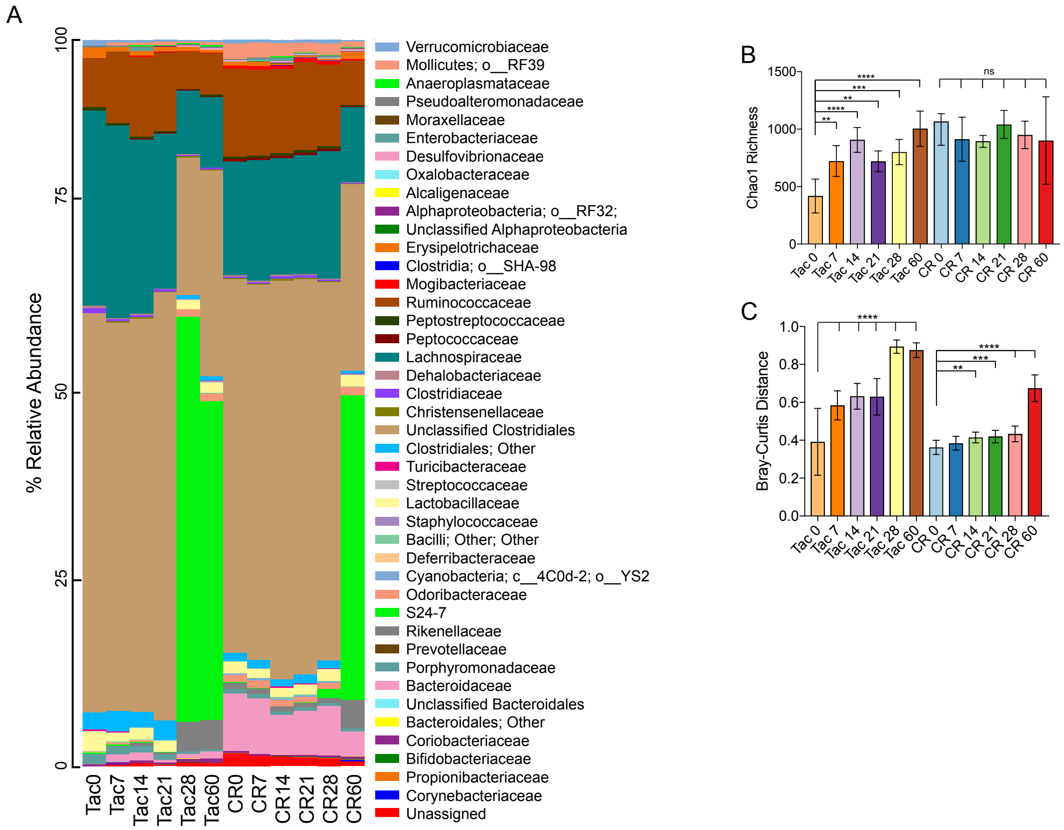

Supplementary Figure 4

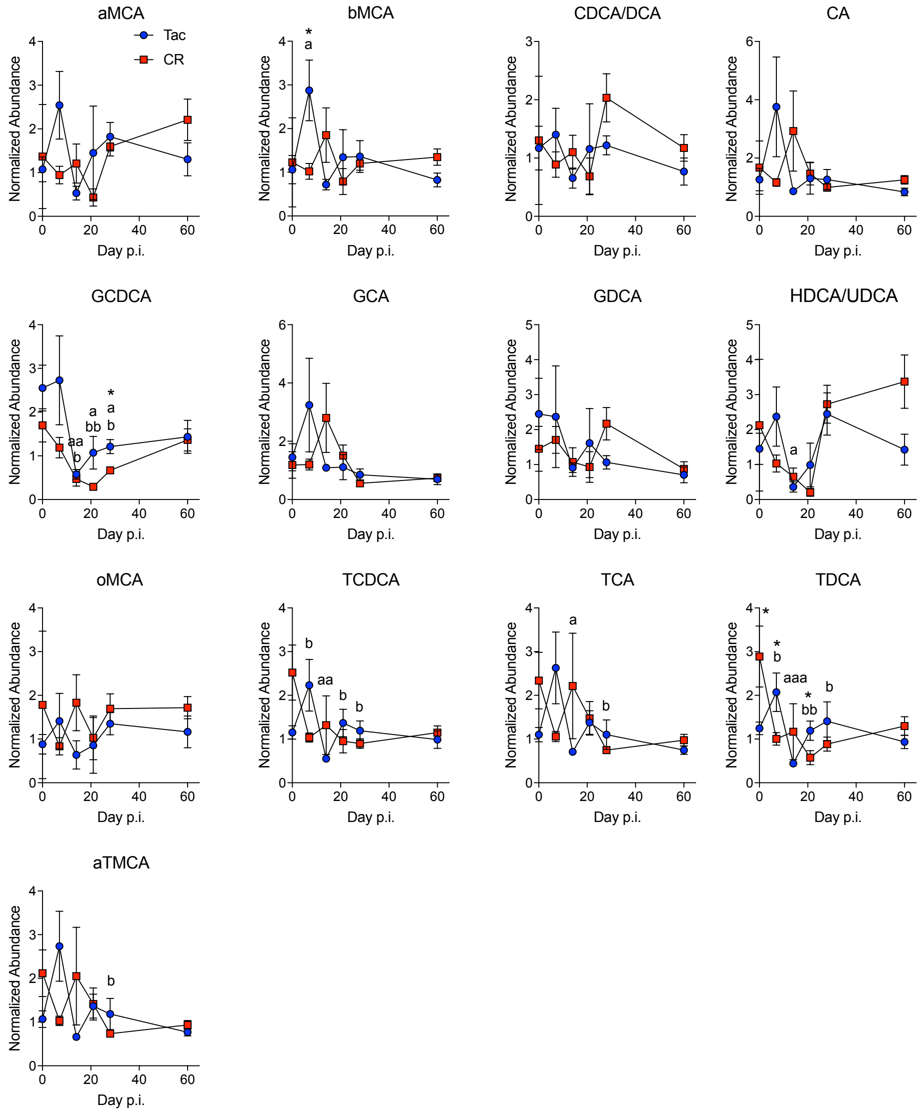

Supplementary Figure 5

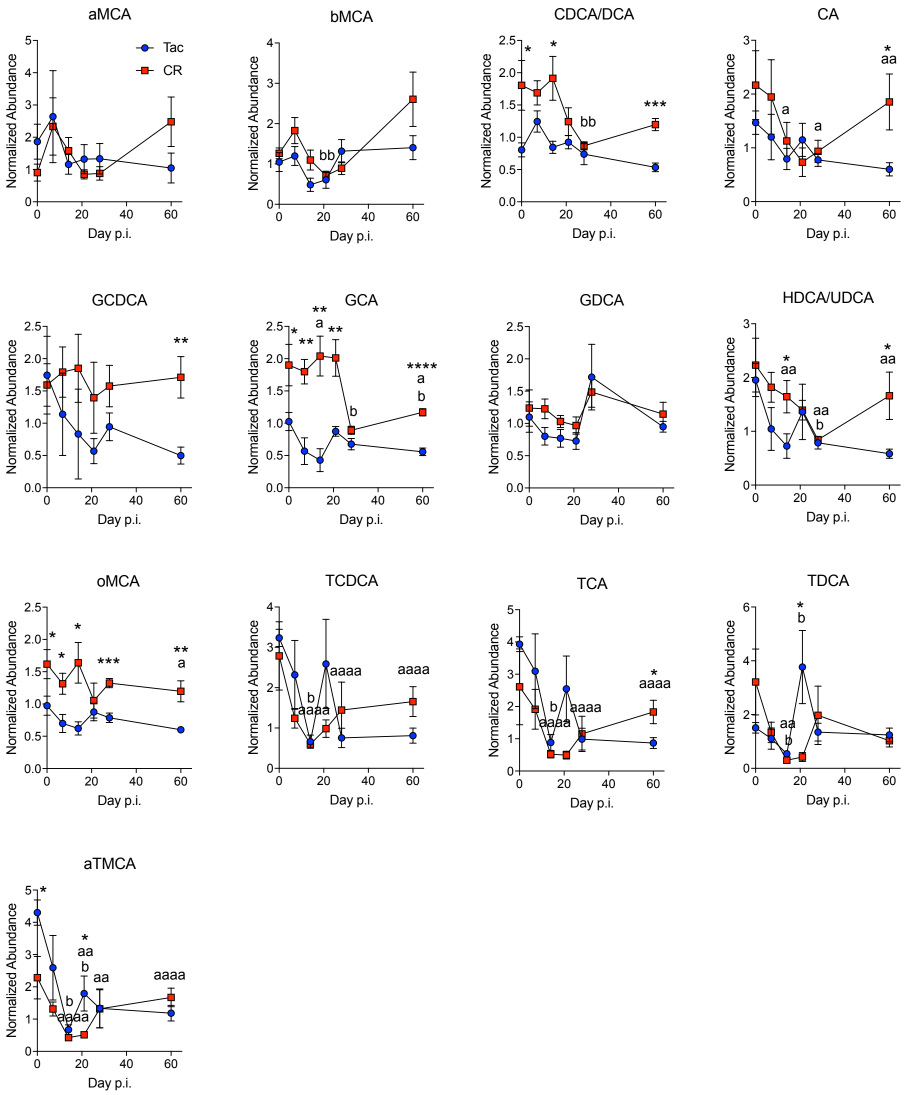

Supplementary Figure 6

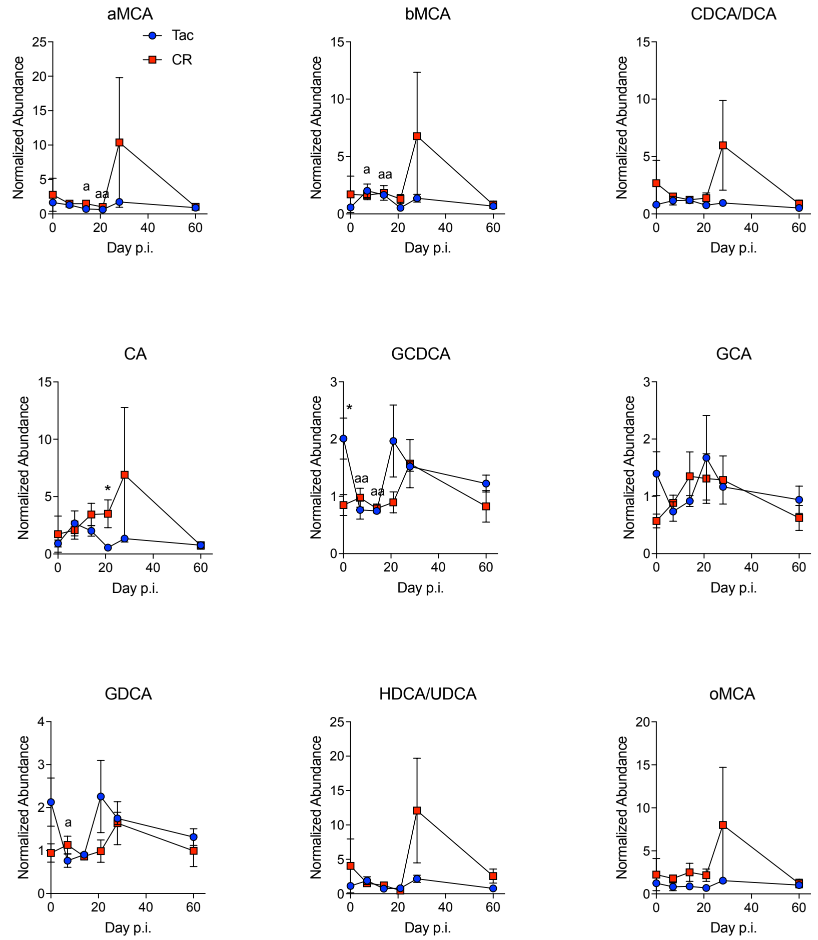

Supplement: Supplementary file 1 — Supplementary Information [file 41598_2019_40266_MOESM1_ESM.pdf]
